# Antitumor Activity of Extract From the Sporoderm-Breaking Spore of *Ganoderma lucidum*: Restoration on Exhausted Cytotoxic T Cell With Gut Microbiota Remodeling

**DOI:** 10.3389/fimmu.2018.01765

**Published:** 2018-07-31

**Authors:** Jiyan Su, Lu Su, Dan Li, Ou Shuai, Yifan Zhang, Huijia Liang, Chunwei Jiao, Zhanchi Xu, Yong Lai, Yizhen Xie

**Affiliations:** ^1^State Key Laboratory of Applied Microbiology Southern China, Guangdong Provincial Key Laboratory of Microbial Culture Collection and Application, Guangdong Institute of Microbiology, Guangzhou, China; ^2^School of Pharmacy and Chemistry, Dali University, Dali, China; ^3^Guangdong Yuewei Edible Fungi Technology Co. Ltd., Guangzhou, China; ^4^School of Pharmaceutical Science, Guangzhou University of Chinese Medicine, Guangzhou, China

**Keywords:** spore of *Ganoderma lucidum*, cytotoxic T cells, exhaustion, immune checkpoints, gut microbiota

## Abstract

As breast cancer is the leading cause of cancer-related deaths in women population worldwide, ongoing endeavor has been made for alternative regimens with improved efficacy but fewer adverse effects. Recently, active components from the spores of *Ganoderma lucidum* have attracted much attention for their versatile biological activities owing to the advance in sporoderm-breaking technology. Here, anticancer potential of an extract derived from the sporoderm-breaking spores of *G. lucidum* (ESG) was explored in a 4T1-breast cancer xenograft mice model. Results showed that ESG was able to suppress 4T1 tumor growth *in vivo* rather than *in vitro*. Flowcytometry analysis revealed that ESG could significantly increase both cytotoxic T cell (Tc) population and the ratio of Tc to helper T cell (Th) in peripheral blood of the tumor-bearing mouse; similar promotion on Tc was also found in tumor-infiltrating lymphocyte. Moreover, ESG evidently downregulated the two immune checkpoints, programmed cell death protein-1 (PD-1, in the spleen) and cytotoxic T lymphocyte antigen-4 (CTLA-4, in the tumor), suggesting that ESG could effectively restore the T cell paradigm by recovering the exhaustion status *via* suppressing the co-inhibitory checkpoints. By 16S rRNA gene sequence analysis on the fecal microbiota, it was found that ESG would remodeling the overall structure of the samples from tumor-bearing mice toward that of the normal counterparts, including 18 genera in 5 phyla, together with regulations on several genes that are responsible for signaling pathways involved in metabolism, cellular processes, and environmental information processing. Collectively, this study demonstrated that ESG would serve as a natural anticancer adjuvant *via* a restoration on the exhausted Tc, highlighting important clinical implications for the treatment of breast cancer.

## Introduction

Breast cancer is one of the most frequently diagnosed cancers and leading causes of cancer-related death both worldwide (1, 2). It is a highly heterogeneous disease. Besides the multiple signaling pathways that mediate its initiation and progression, accumulating epidemiology studies have proposed several suspected risk factors for breast cancer, including exposures to cancerigenic substances, certain lifestyles (such as lack of physical exercise, intemperance), aging and family history (3). To date, surgical resection, adjuvant chemotherapy, radiotherapy, and hormone therapy represent the main treatment options for early-stage breast cancer (4), but they are still unsatisfied due to the emerging drug resistance and adverse toxic effects (5, 6). Even more disturbing, triple-negative breast cancer does not respond to hormone therapies (7).

Despite the slow progress in the above regimes, recent success of cancer immunotherapy utilizing immune checkpoint blockade has reformed the treatment algorithms in various aggressive neoplastic diseases (8, 9). Immunotherapy is based upon the “3E immunoediting theory” (Eliminate, Equilibrium, and Escape) (10). It reminds that immune surveillance stands at the very heart of the fighting against cancer, but T cells would be exhausted and have decreased effector function and proliferative capacity during cancer progression, due the overexpression of immune checkpoints, and the interaction with their ligands (11, 12). So far, immunotherapy targeting programmed cell death protein-1 (PD-1)/programmed death-ligand 1 (PD-L1) has been on it way of clinical trials (13) for breast cancer, although optimal selection of ideal candidates to the immune therapy remains a challenge. On the other hand, increasing evidence highlights the cardinal role of gut microbiota in tumour genesis (14, 15) and that in the outcomes of chemotherapy (16) and immunotherapy (17, 18), due to their intrinsic capacity of drug metabolism and the influence on host metabolizing homeostasis (19, 20).

*Ganoderma lucidum* (Leyss. ex Fr.) Karst. is a valuable medical macrofungi that has long been used in traditional Chinese medicine for health and longevity for thousands of years (21). In addition to numerous benefit for the treatment of allergy, cardiovascular diseases, diabetics (22, 23), ample evidence have revealed that *G. lucidum* exerts anticancer effects not only *via* direct cytotoxicity approaches, such as cell cycle arrest (24), apoptosis induction (25), and migration inhibition (26), but also, more importantly, through several ways of immune enhancement (27–29). Recently, although active components from fruiting body or mycelia are still hotspots of *G. lucidum* studies, those from the spores of *G. lucidum* (SG) have attracted much attention for their versatile biological activities owing to the advance in sporoderm-breaking technology. Studies reported that SG displayed anticancer potentials against Sarcoma 180 and HCT116 (30–32). Moreover, it has been found that polysaccharide content in SG was higher than that in fruiting body (33) and that sporoderm-breaking would potentiate the immunoregulation activity of spore (32, 34, 35). These studies suggest that SG may serve as promising anticancer agent for cancer therapy. In this study, anticancer potential of an extract from the sporoderm-breaking SG, for the first time, was investigated in a breast cancer xenograft mice model. The exploration focused on the interaction between T cell restoration and gut microbiota, to make a comprehensive interception for the anticancer activity of the sporoderm-breaking SG.

## Materials and Methods

### Animals

BALB/c mice (female, 18–22 g, aged 6–8 weeks) were provided by Guangdong Medical Laboratory Animal Center (Guangzhou, Guangdong, China). All animals were housed at 20 ± 2°C with a humidity of 50 ± 5% in a 12 h light/dark cycle with food and water *ad libitum*. The animals were acclimatized for 7 days, and the experiment was performed according to the Guidelines of Guangdong Institute of Microbiology Laboratory Animal Center, Guangdong Institute of Microbiology Laboratory Animal Ethics Committee. The experimental protocols were approved by the Guangdong Institute of Microbiology Laboratory Animal Ethics Committee.

### Preparation for Extract of the Sporoderm-Breaking Spore of *G. lucidum* (ESG)

The sporoderm-breaking SG was provided by Guangdong Yuewei Edible Fungi Technology Co. Ltd. It was extracted with boiling water (15 L/kg) for 2 h and concentrated under vacuum. The concentrated extract was subjected to two to three cycles of precipitation with anhydrous ethanol at a final ethanol percentage of 85%. The obtained precipitate was dissolved in water and dialyzed with 3,500 Da dialysis tube (MWCO). Content in the 3,500 Da dialysis tube was then dialyzed in 100 kDa dialysis tube (MWCO). The dialysate was pooled, concentrated, and lyophilized, to obtain ESG with a yield of 0.4%.

Characteristics analysis for ESG was performed as described by Qiao et al. (36) with mild modification. Results showed that sugar content of ESG was 50% (determined by the phenol–sulfuric acid method using glucose as standard), while protein was hardly detected by BCA methods with the BCA protein kit (Kang wei shiji Co. Ltd., Beijing, China), suggesting that ESG is rich in polysaccharide. Weight average molecular weight (*M*_w_) by size-exclusive high performance liquid chromatography showed that polysaccharide in ESG was about 3.6 kDa (Table S1 and Figure S1 in Supplementary Material). Monosaccharide composition analysis by gas chromatograph showed that ESG was mainly made up of glucose (Figure S2 in Supplementary Material).

### Cell Culture

Murine metastatic breast cancer 4T1-cell line was obtained from the Cell bank of Chinese Academy of Sciences, Shanghai, China. 4T1 cells were cultured in high glucose DMEM medium (4.5 mg/mL, Gibco, NY, USA) supplemented with 10% fetal bovine serum (Gibco, NY, USA) and 1% penicillin/streptomycin (Gibco, NY, USA) and maintained in humidified incubators at 37°C under an atmosphere of 5% CO_2_.

### Cytotoxicity Assay

4T1 cells were seeded in a 96-well plate at a density of 1.25 × 10^4^ cells/mL (sextuple wells in each group) and treated with ESG in complete DMEM medium at multiple concentrations (12.5, 25, 50, 100, and 200 µg/mL) for 24 and 48 h. Then, the medium was replaced with 100 µL of complete DMEM medium containing 0.5 mg/mL 3-(4,5-dimethyl-2-thiazolyl)-2,5-diphenyl-2-H-tetrazolium bromide (MTT) for another 4-h incubation. At last, the medium was discarded, and 150 µL of DMSO was added to dissolve the formazan. The optical density was measured at 490 nm on a microplate reader.

### 4T1-Breast Cancer Xenograft Model Induction

To induce the breast cancer xenograft model, female BALB/c mice were implanted with 4T1 tumor cells by subcutaneous injection at the right forleg armpit (0.1 mL/mouse, 2 × 10^5^ cells/mouse), and then they were randomly divided into Model group, paclitaxel group (PTX, Hainan Quanxing Pharmaceutical Co. Ltd., Hainan, China), and the ESGH group (400 mg/kg), ESGL group (200 mg/kg), eight for each. On the same day, control animals (Normal group, eight mice) received an injection of 0.1 mL of complete DMEM medium at the similar site. Over the following 21 days, mice in the PTX group were administrated with PTX at a dose of 15 mg/kg (i.p.) twice a week; mice of the ESG groups were given different doses of ESG (i.g.) every day. Normal group and Model group received equal volume of water.

### Tumor Measurement and Histology Observation by Hematoxylin–Eosin Staining

Throughout the 21-day administration, tumor volume was measured with an electronic vernier caliper every 3 days since sixth day. Tumor volume was calculated as *V* = *a* × *b*^2^/2, where *a* indicates the longer diameter and *b* indicates the shorter diameter. 24 h after the last administration, all animals were sacrificed by cervical dislocation. Tumors, spleens, and peripheral blood were harvested for further analysis.

Tumors were weighted, photographed, cut into several segments, and then stored according to different purposes once they were harvested. One segment of the tumor was fixed in 4% neutral formalin (in PBS), embedded by paraffin, and stained with hematoxylin–eosin (HE). The stained sections were observed and photographed under a light microscope (with 200× magnification).

### Peripheral Blood Lymphocyte Analysis by Flow Cytometry

Peripheral blood was collected from the orbital vein plexus with EDTA-Li micro-anticoagulant tube. 50 µL of blood was stained with FITC anti-mouse CD3 (0.125 μg/test), APC anti-mouse CD4 (0.0625 μg/test), PE anti-mouse CD8 (0.125 μg/test, Invitrogen, Thermo Fisher Scientific, San Diego, CA, USA) at 4°C in dark for 30 min, and then erythrocytes were lysed in ACK Lysis Buffer for 10 min. Following by two washes with pre-cold PBS, T cell subsets in the peripheral blood were enumerated with a FACS Canto II cytometer (BD, NY, USA), and the data were analyzed by Diva software (version 6.1.3).

### Tumor-Infiltrating Lymphocyte (TIL) Isolation and Analysis

Tumor segment kept in cold PBS was used for TIL isolation and analysis. In brief, they were minced and digested in 3 mL digestive medium, which was mainly composed of basic RPMI-160 medium supplemented with 0.1% Type IV collagenase (Invitrogen, Thermo Fisher Scientific, Grand Isle, NY, USA), 350 U/mL DNAse I (Roche, Basel, Switzerland), and 1% penicillin–streptomycin. Then, it was ground in pre-cold PBS by passing through a 70 µm strainer, washed with PBS, and resuspended in basic RPMI-160 medium. TILs from the obtained cell suspension were separated with Mouse 1× Lymphocyte Separation Medium (Dakewe Biotechnology Co. Ltd., Shenzhen, China) according to the manufacture’s instruction. TILs were stained with FITC anti-mouse CD3 (0.125 μg/test), APC anti-mouse CD4 (0.0625 μg/test), and PE anti-mouse CD8 (0.125 μg/test) at 4°C in dark for 30 min. After two washes with pre-cold PBS, T cell subsets in TIL were enumerated with a FACS Canto II cytometer, and the data were analyzed by Diva software (version 6.1.3).

### Programmed Cell Death Protein-1 (PD-1) Protein Content Determination by ELISA

About 100 mg of spleen tissue was cut into pieces, homogenized in ice-cold PBS with proteinase inhibitor cocktail (Roche, USA) for 30 s, centrifuged at 18,000 × *g* for 20 min at 4°C. Protein content in the supernatant was quantified with BCA protein kit (Kang wei shiji Co. Ltd., Beijing, China). PD-1 protein content in spleen was measured using the Mouse PD1 ELISA Kit (Abcam, Cambridge, UK, Cat: ab210971) according to the manufacturer’s instruction.

### Immunohistochemistry (IHC) for Cytotoxic T Lymphocyte Antigen-4 (CTLA-4)

Immunohistochemistry for CTLA-4 was performed with the formalin-fixed, paraffin-embedded tumor segment. Briefly, the slides were deparaffinized. Antigen retrieval was carried out by incubation in EDTA buffer (pH 8.0) *via* microwave heating. After being washed with PBS, endogenous peroxidase in the section was blocked with 3% H_2_O_2_ in dark for 25 min. Sections were blocked with 3% normal non-immune serum and then incubated with primary antibody against mouse CTLA-4 (1:100, Lifespan Biosciences, London, UK) at 4°C overnight, and then with HRP-conjugated secondary antibodies at room temperature for 50 min. Finally, the sections were stained with DAB substrate and counterstained with hematoxylin. The mean density of positive area was calculated as ratio of integrated optical density to the total pixel of each picture (IOD/10^6^ pixel), which was analyzed by Image Pro Plus 6.0 software (Media Cybernetics, Silver Spring, MD, USA).

### Total RNA Extraction and Quantitative Real-Time PCR

Total RNAs from spleen and tumor were extracted with TRIzol according to the manufacturer’s instructions (Invitrogen, Thermo Fisher Scientific, Grand Isle, NY, USA). 0.1 μg of total RNA was reverse transcribed using the PrimeScript™ RT reagent kit (Takara Bio, Inc., Shiga, Japan) following the supplier’s protocol. The reactions were incubated at 35°C for 15 min, then at 85°C for 5 s, and the products were stored at 4°C. The PCR primer sequences are listed in Table 1. Real-time PCR reactions were performed with SYBR^®^ Premix Ex Taq™ II (Takara Bio, Inc., Shiga, Japan), and the reaction program in an StepOnePlus Real-Time PCR system (Thermo Fisher Scientific, Grand Isle, NY, USA) was as follows: a precycling stage at 95°C for 30 s, then 40 cycles of denaturation at 95°C for 5 s and annealing at 60°C for 30 s. Fluorescence was measured at the end of each annealing step, and the melting curves were monitored to confirm the specificity of the PCR products. The 2^−ΔΔCt^ method was used to determine the mRNA expression levels of *pd1* and *ctla4* relative to control gene *gapdh*.

**Table 1:** Primers for real-time PCR.

Gene name	GenBank accession.		Primer	Product length (bp)
*pd1*	NM_008798.2	Sense	TTTGAGCCAACCCGTCCAGGAT	90
		Antisense	CGCCGTGTGTCAAGGATGTTCA	
*ctla4*	NM_001281976.1	Sense	GAGGTCTGTGCCACGACATTCA	190
		Antisense	CGTTGCCCATGCCCACAAAGTA	
*gapdh*	NM_008084	Sense	AAATGGTGAAGGTCGGTGTGAAC	90
		Antisense	CAACAATCTCCACTTTGCCACTG	

### 16S rRNA Gene Sequence Analysis of Gut Microbiota in Fecal Samples

Sequencing service was provided by Personal Biotechnology Co., Ltd., Shanghai, China. Total DNA was isolated from the fecal samples as previously reported with some modification (37). Briefly, OMEGA Soil DNA Kit (OMEGA, US) was used following the manufacturer’s recommendations. The bacterial 16S rRNA gene V3–4 region was amplified by PCR using the forward primer (5′-AYTGGGYD TAAAGNG-3′) and the reverse primer (5′-TACNVGGGTATCTAATCC-3′). The PCR products were separated by gel electrophoresis and purified using the AP-GX-500 DNA Gel Extraction Kit (Axygen, Corning, USA). Library was build up with the obtained products and then sequenced on a MiSeq sequencing platform (Illumina, USA) as described by Zhao et al. (38).

### Bioinformatics Analysis

The trimmed and assembled sequences from each sample were aligned to the Greengene 16S rRNA database set 10 using the best hit classification option to classify the taxonomy abundance in QIIME^1^ (39). Bacterial operation taxonomic units (OTU) were generated using the uclust function in QIIME.^2^ A Venn diagram was generated to compare OTUs between groups. The following statistics were performed by R software. ACE, Chao, Simpson, and Shannon indices were calculated for α-diversity evaluation. Principal component analysis (PCA) and UniFrac distance-based Nonmetric Multidimensional Scaling (NMDS) were employed to assess β-diversity. Hierarchical clustering analysis of the OTUs presented by heatmap was performed using the heatmap package v1.0.7 running in R v3.2.1.^3^ Taxon-based analysis and LefSe analysis were applied to identify different taxa microbes among lines using the default parameters (40).

Microbial functions were predicted using PICRUSt (41). The OTUs were mapped to gg13.5 database at 97% similarity by QIIME’s command “pick_closed_otus.” The OTUs’ abundance was normalized automatically using 16S rRNA gene copy numbers from known bacterial genomes in Integrated Microbial Genomes. The predicted genes and their functions were aligned to Kyoto Encyclopedia of Genes and Genomes (KEGG) database, and differences among groups were compared through software STAMP^4^ (42). Two-side Welch’s *t*-test and the Benjamini–Hochberg FDR (*p* < 0.05) correction were used in two-group analysis.

### Statistics

Statistical analysis was performed with SPSS 22 (IBM Corp., NY, USA). Datasets from each experiment were subjected to normal distribution test first. If according with the normal distribution, the data was analyzed by one-way analysis of variance (ANOVA), following by pairwise comparison with different parametric test depending on test for homogeneity of variance, otherwise the data was compared by Kruskal–Wallis *H* Test. In ANOVA, *post hoc* LSD test was applied for difference analysis under homogeneity of variance, if not, a Dunnett’s test would be applied. ^#^*p* < 0.05 and ^##^*p* < 0.01 as compared with Normal group; *p < 0.05 and **p < 0.01 as compared with Model group.

## Results

### ESG Inhibited Tumor Growth

**Figure 1:**
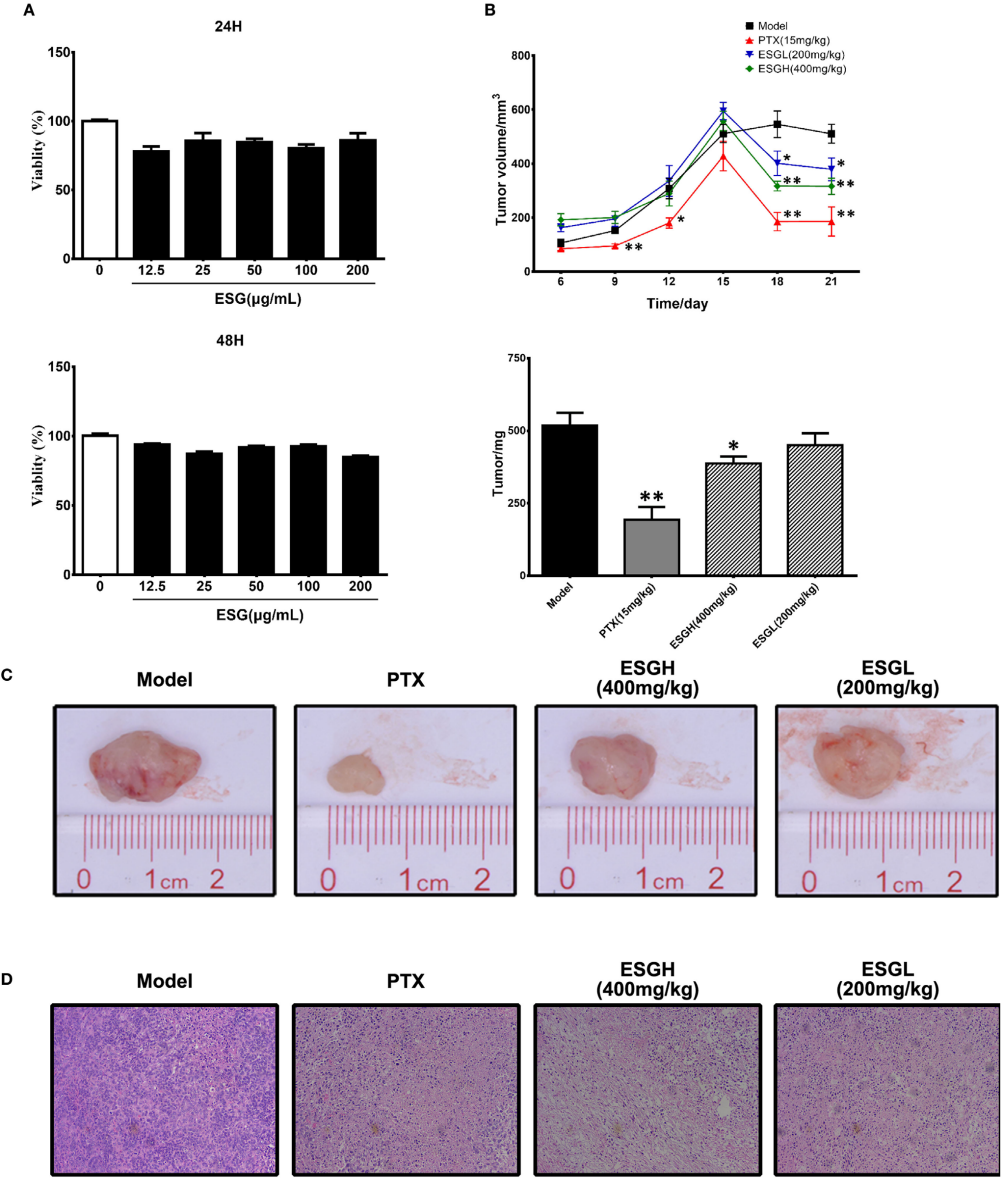
Effect of ESG on 4T1-xenograft model. **(A)** Effect of ESG on the viability of 4T1 cell *in vitro* (48 and 72 h). **(B)** Tumor volume changes and tumor mass. **(C)** Representative images for tumor from each group. **(D)** Representative hematoxylin–eosin staining images for tumor tissue from each group (200× magnification). Values were represented the means ± SEM (*n* = 6–8). *p < 0.05 and **p < 0.01 versus Model group.

In Figure 1A, it was found that viability of 4T1 cells was not affected by either 24 or 48 h treatment of ESG (12.5–200 μg/mL) *in vitro*, implying that the antitumor activity of ESG was not mediated by a directly cytotoxicity. However, ESG exhibited a favorite antitumor activity in the xenograft model. Two mice of the PTX group died during the experiment. As shown in Figures 1B,C, tumor volume of Model group kept increasing throughout the 21-day experiment. By comparison, tumor volumes of the PTX group and ESG groups (400 and 200 mg/kg) showed remarkable reduction since the 15th day, although they also kept increasing in the earlier days. Finally, tumors of the Model group weighted 512 ± 45 mg, while those of PTX group and ESG groups (400 and 200 mg/kg) were significantly decreased to 193 ± 45 mg (*p* < 0.01), 387 ± 23 mg (*p* < 0.05), and 450 ± 41 mg, respectively, which were in accordance with the depressed volume. Moreover, histology analysis by HE staining revealed that tumors from Model group exhibited a homogeneous distribution of viable cells, while those of PTX group and ESG groups showed significant indication of necrosis, including shrinkage of the cells, nuclear condensation, and fibrosis (Figure 1D).

### ESG Recovered Proportions of Tc Cells Both in Peripheral Blood and Tumor Microenvironment (TME)

**Figure 2:**
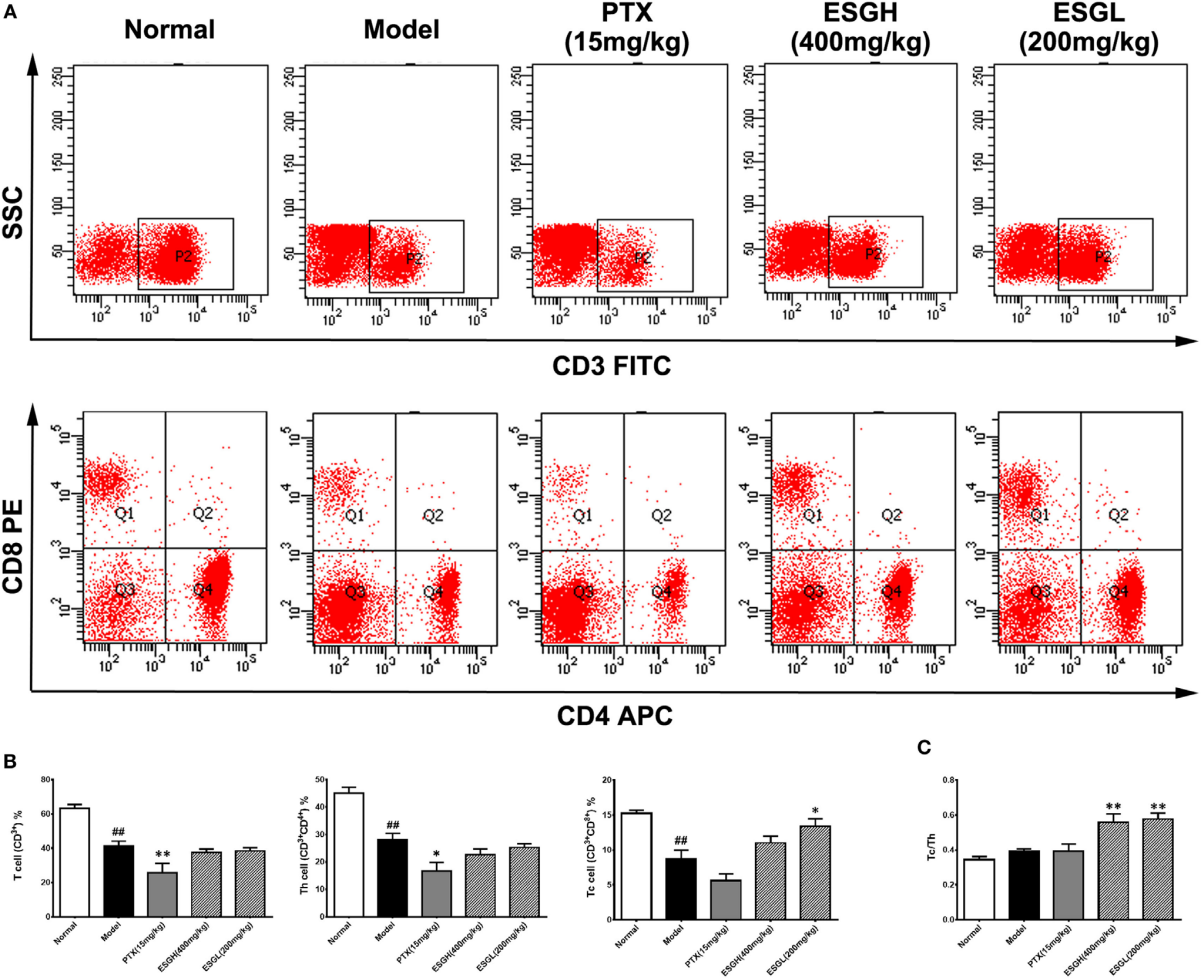
Effect of ESG on T cell subsets in peripheral blood. **(A)** Representative scatter diagram. **(B)** Quantitative analysis for T cell subsets in peripheral blood. **(C)** Ratio of Tc/Th in peripheral blood. Values were represented the means ± SEM (*n* = 6–8). ^#^*p* < 0.05 and ^##^*p* < 0.01 versus Normal group; *p < 0.05 and **p < 0.01 versus Model group.

Since the antitumor activity of ESG was independent of cytotoxicity, we analyzed the impact of oral administration of ESG on immune surveillance system by flowcytometry (FCM) as it plays a cardinal role in the suppression of tumor. Results showed that in peripheral blood (Figure 2), percentage of T cell (CD^3+^) of Model group was obviously lower than those of Normal group (*p* < 0.01), and distinct decreases were also found in the proportions of the two main subsets (*p* < 0.01), helper T cell (Th, CD3^+^CD4^+^) and cytotoxic T cell (Tc, CD3^+^CD8^+^). PTX made a more serious lost on T cell and Th cell when comparing with those of Model group (*p* < 0.01 and *p* < 0.05, respectively). On the other hand, although failing to lift the amount of T cell or that of the Th subset, ESG (200 mg/kg) prominently increased the percentage of Tc cell in the circulatory system (*p* < 0.05). Moreover, the ratio of Tc/Th was obviously elevated after ESG treatment (400 and 200 mg/kg, *p* < 0.01).

**Figure 3:**
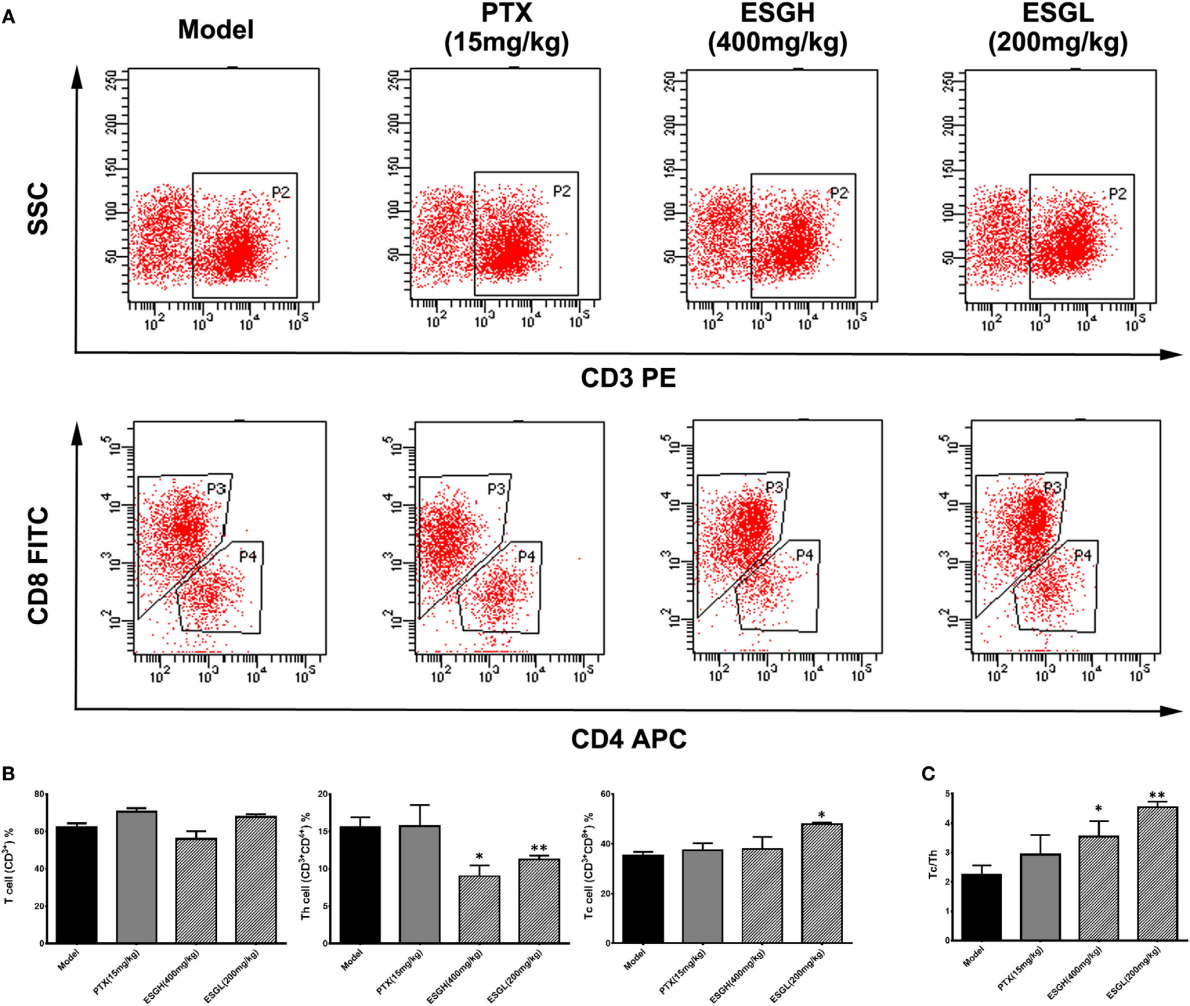
Effect of ESG on T cell subsets in tumor-infiltrating lymphocyte (TIL). **(A)** Representative scatter diagram. **(B)** Quantitative analysis for T cell subsets in TIL. **(C)** Ratio of Tc/Th in TIL. Values were represented the means ± SEM (*n* = 6–8). *p < 0.05 and **p < 0.01 versus Model group.

To make a more detail observation for the immune surveillance system in the TME, TIL was also determined by FCM. It was found that, although percentage of Th cell was decreased (*p* < 0.01), ESG brought out an evident increase on the Tc cell proportion in the TME (*p* < 0.05) accompanying with a higher Tc/Th ratio (*p* < 0.01), which was quite corresponding to the results of peripheral blood (Figure 3). Nevertheless, this promotion on Tc cell by ESG did not exist when ESG directly acted on splenocytes *in vitro* (Figure S3 in Supplementary Material).

### ESG Suppressed the Immune Checkpoints

**Figure 4:**
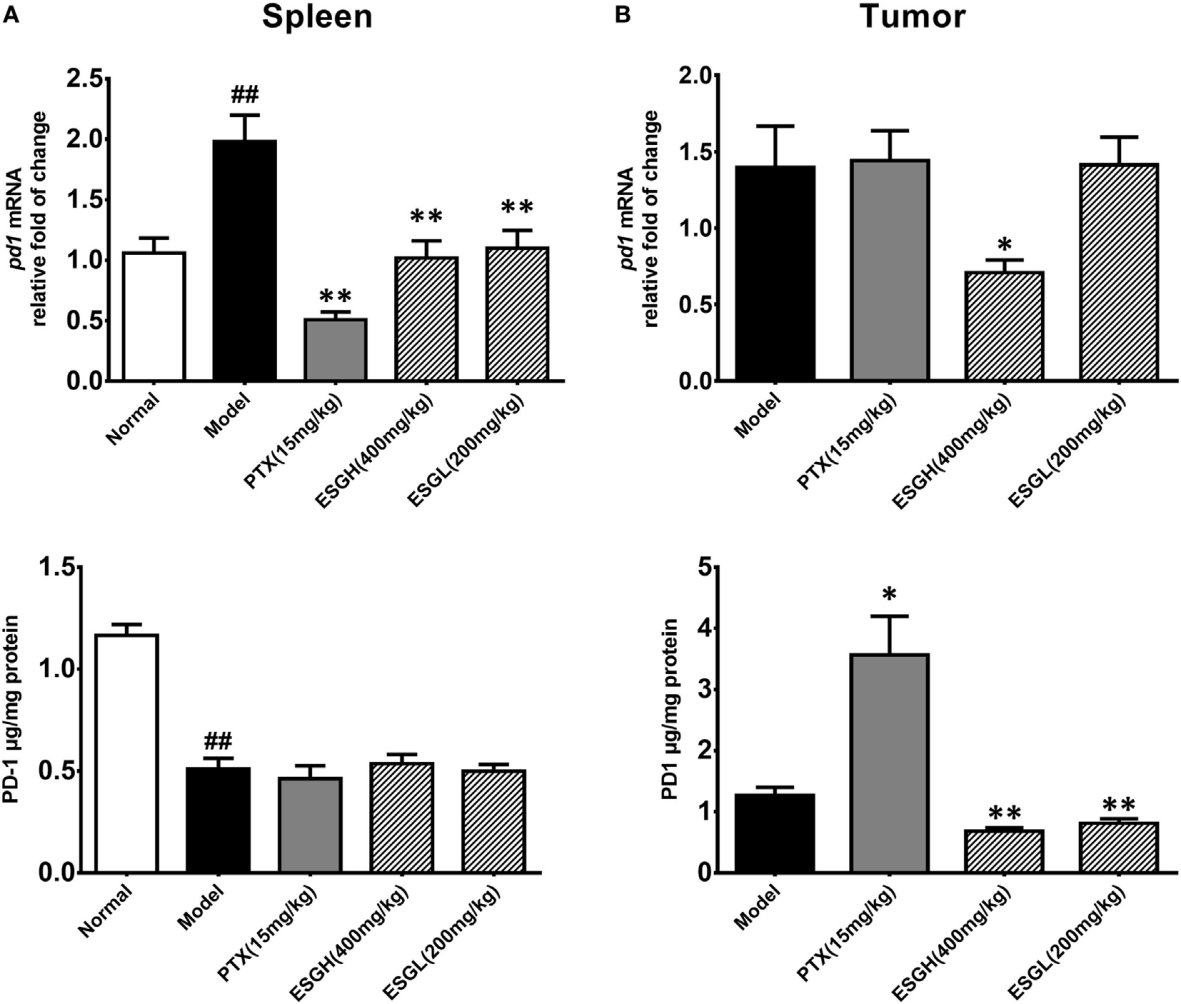
Effect of ESG on PD-1 in spleen **(A)** and tumor **(B)**. The upper panels are results of quantitative real-time PCR for *pd1*. The lower panels are results of ELISA for PD-1 protein. Values were represented the means ± SEM (*n* = 6–8). ^#^*p* < 0.05 and ^##^*p* < 0.01 versus Normal group; *p < 0.05 and **p < 0.01 versus Model group.

To further evaluate the enhancement of ESG on the T cell status, expressions of the two immune checkpoints, PD-1 and CTLA-4, were explored. Results from Figure 4 displayed that 4T1-cell xenograft significantly upregulated the mRNA expressions of *pd1* in spleen (*p* < 0.01), while decreasing the protein level of PD-1 (*p* < 0.01). In comparison, both PTX and ESG obviously downregulated the *pd1* gene expression (*p* < 0.01), but not affecting the protein level of PD-1. In tumor tissue, PTX evidently increased PD-1 protein expression (*p* < 0.05), while the mRNA level was almost the same as those from the Model group. By contrast, ESG exhibited obvious inhibition on PD-1, especially that the 400 mg/kg ESG made strong suppressions on both levels of mRNA and protein (*p* < 0.05 and *p* < 0.01, respectively).

**Figure 5:**
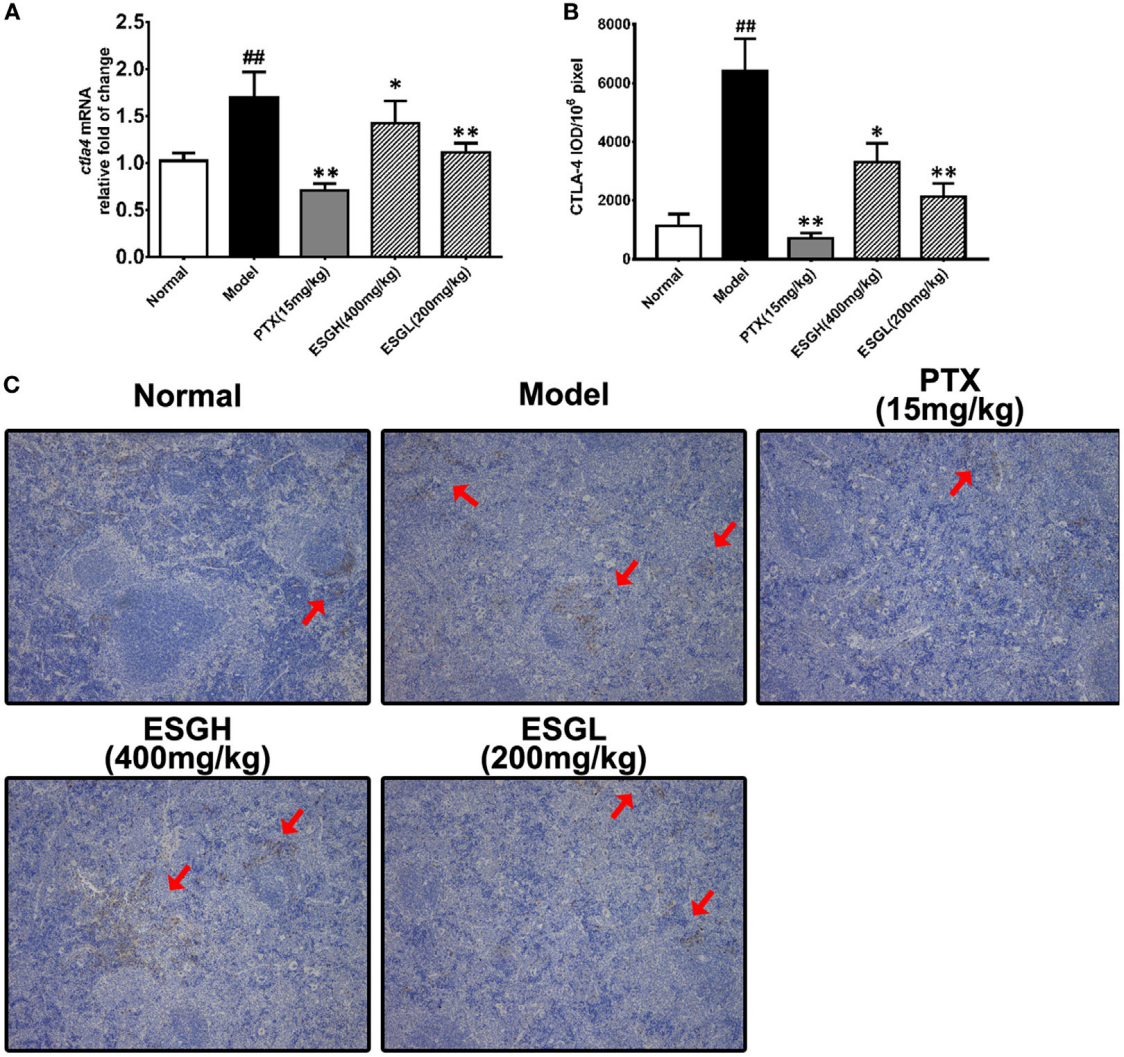
Effect of ESG on cytotoxic T lymphocyte antigen-4 (CTLA-4) in spleen. **(A)** Quantitative real-time PCR for *ctla4*. **(B)** Immunohistochemistry (IHC) for CTLA-4 in spleen. The mean density of positive area was calculated as ratio of integrated optical density to the total pixel of each picture (IOD/10^6^ pixel). **(C)** Representative IHC image (400×) for CTLA-4 in spleen. Values were represented the means ± SEM (*n* = 6–8). ^#^*p* < 0.05 and ^##^*p* < 0.01 versus Normal group; *p < 0.05 and **p < 0.01 versus Model group.

**Figure 6:**
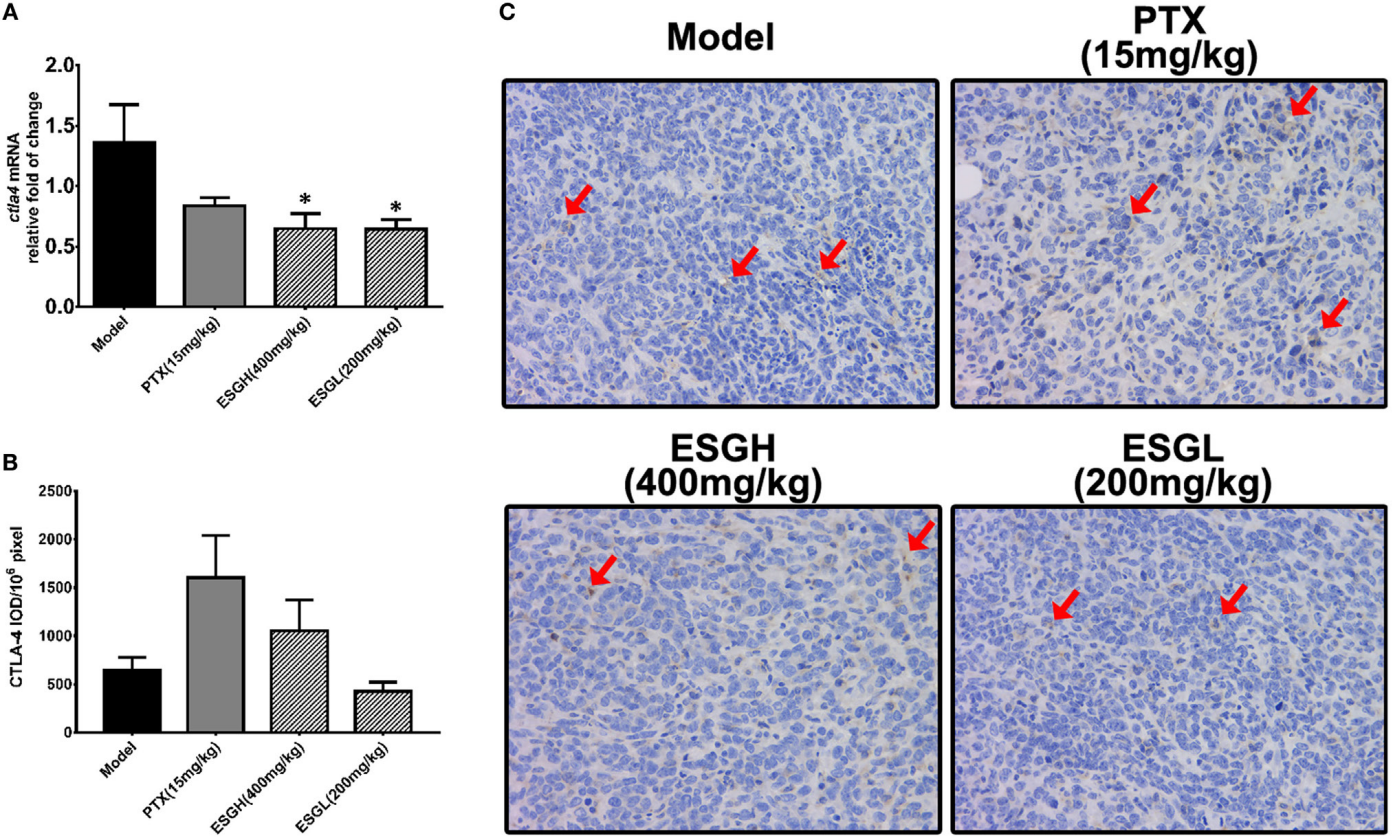
Effect of ESG on cytotoxic T lymphocyte antigen-4 (CTLA-4) in tumor. **(A)** Quantitative real-time PCR for *ctla4*. **(B)** Immunohistochemistry (IHC) for CTLA-4 in tumor. The mean density of positive area was calculated as ratio of integrated optical density to the total pixel of each picture (IOD/10^6^ pixel). **(C)** Representative IHC image (400×) for CTLA-4 in tumor. Values were represented the means ± SEM (*n* = 6–8). *p < 0.05 and **p < 0.01 versus Model group.

Regarding to CTLA-4, tumor xenograft made an evident upregulation on mRNA and protein level in spleen, while both treatments of PTX and ESG decreased them significantly (Figure 5). In tumor (Figure 6), PTX was intended to decrease mRNA level of *ctla4* but to increase its protein expression. By contrast, both dosages of ESG were able to evidently downregulate *ctla4* mRNA (*p* < 0.05), whereas not affecting the protein expression of CTLA-4 apparently.

### ESG Modulated the Gut Microbiota

#### Overall Structural Modulation of Gut Microbiome After ESG Treatment

**Figure 7:**
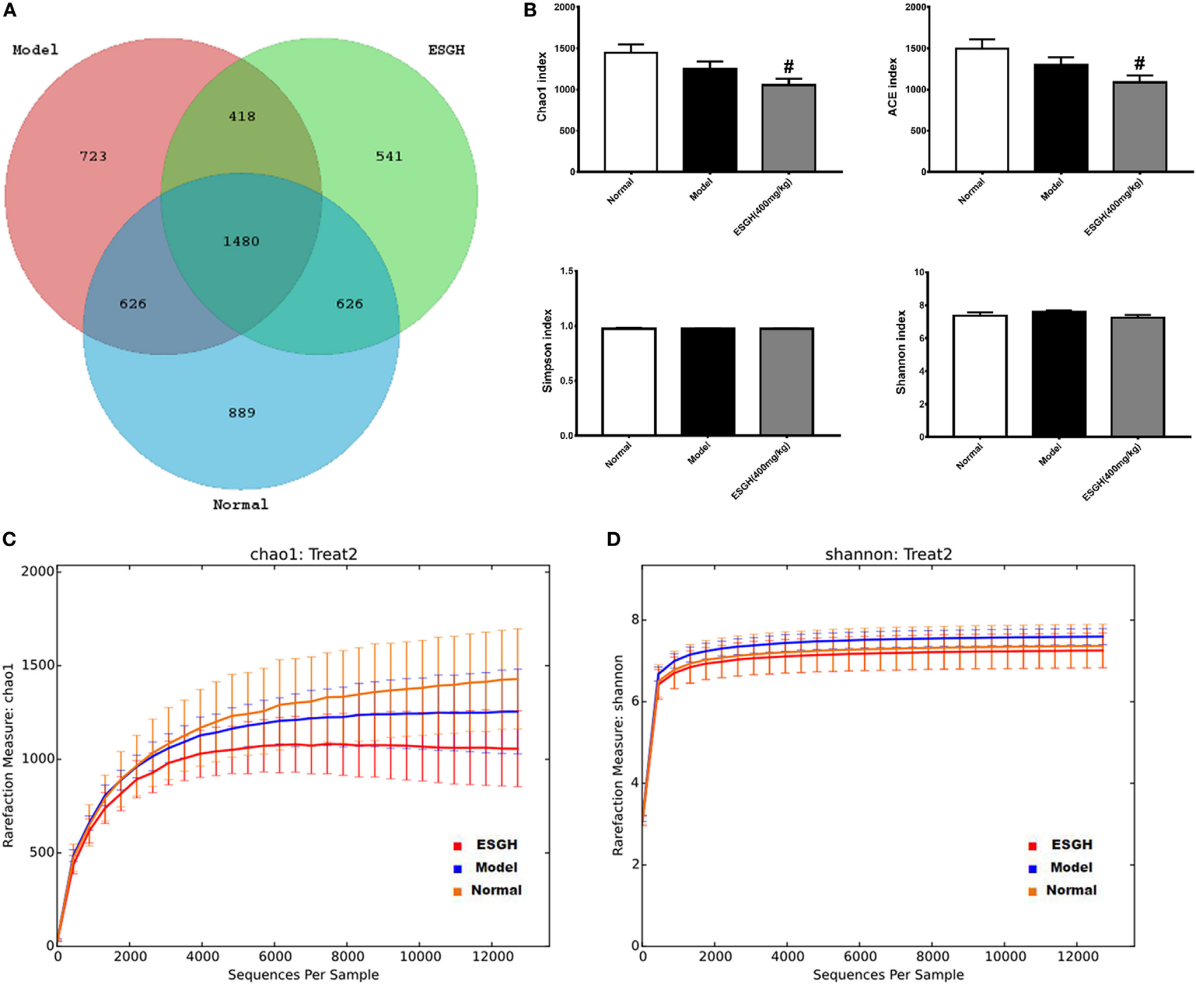
α-Diversity analysis on the fecal microbiota. **(A)** Venn diagram. **(B)** Alpha diversity indices comparison. **(C,D)** Rarefaction curve. Values were represented the means ± SEM (*n* = 8). ^#^*p* < 0.05 and ^##^*p* < 0.01 versus Normal group; *p < 0.05 and **p < 0.01 versus Model group.

Given that gut microbiota has been recognized as a pivotal assistant in chemotherapy and immunotherapy, we investigated its possible involvement in the efficacy of ESG in this part. As the above data indicated that ESGH group (400 mg/kg) exhibited better antitumor effect in the 4T1-xenograft model, gut microbiota in fecal samples from the Normal group, Model group, and ESGH group were analysis by the Illumina Miseq sequencing system. A total of 903,411 sequences were obtained from all the fecal samples, with an average of 34,962 sequences per sample (33,526–43,436 sequences). The high-quality sequences were then delineated into 25,394 OTUs at a similarity cutoff of 97% as previously reported (43). Common OTU analysis presented by Venn diagram indicated that there existed 889 unique OTUs in Normal group, 723 in Model group, and 541 in ESGH group (400 mg/kg), respectively, while 1,480 common OTUs were identified in all samples (Figure 7A).

Microbiota community diversity was first evaluated by α-diversity analysis employing indices including Chao1, ACE, Shannon, and Simpson. Chao1 and ACE indices are estimators for community richness (44, 45). Shannon and Simpson indices represent community diversity and uniformity (46). As depicted by the data, gut microbiome of the ESGH group demonstrated significant reduced richness with lower Chao1 and ACE indexes (*p* < 0.01), compared with that of the Normal group (Figure 7B), while overall diversity was not affected. Rarefaction curve (Figures 7C–D) also presented a significant difference in the richness (Chao1), but not diversity (Shannon), among the three groups in this study.

**Figure 8:**
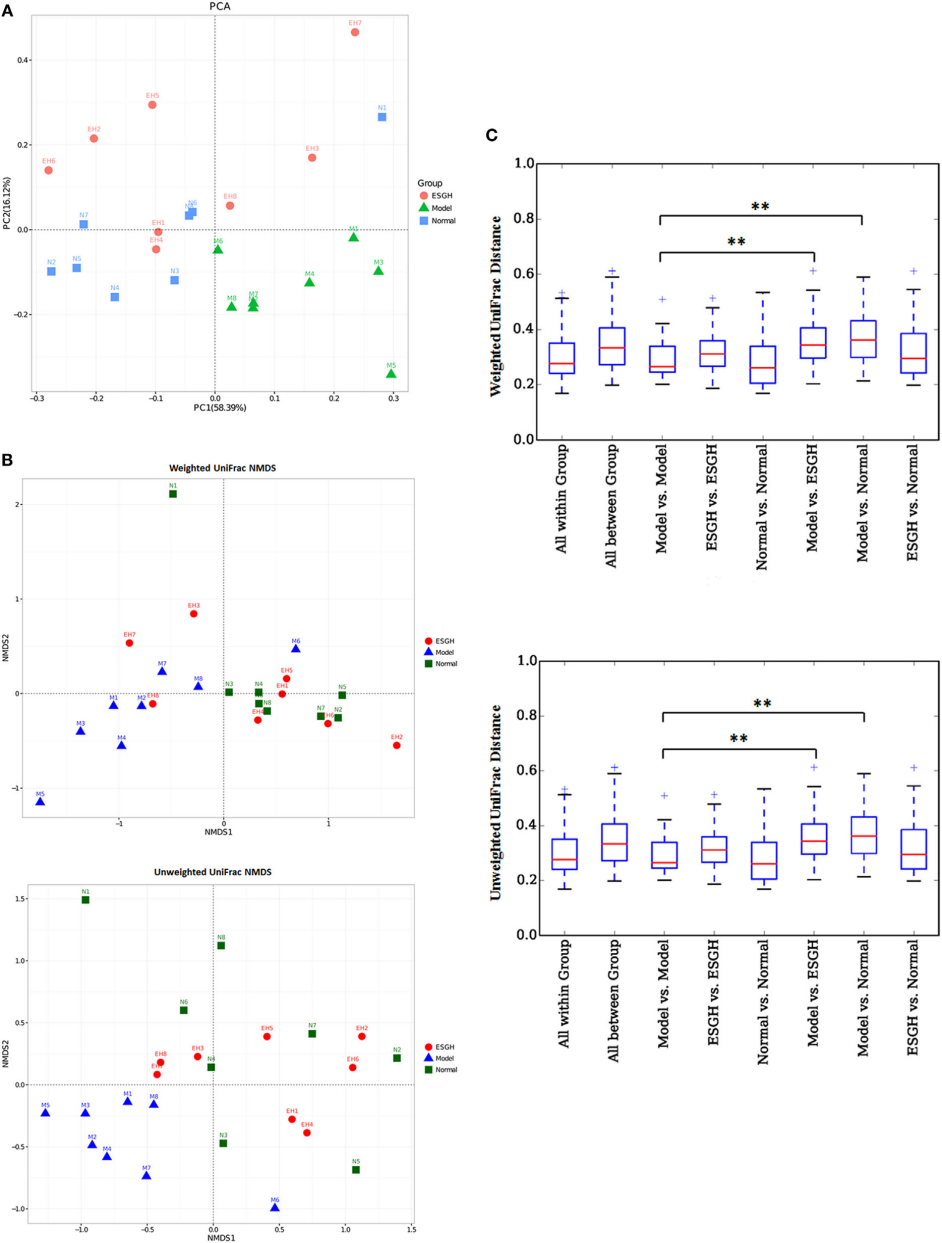
β-Diversity analysis on fecal microbiota. **(A)** Principal component analysis. The percent variation explained by each principal coordinate was indicated on the axes. **(B)** UniFrac distance-based Nonmetric Multidimensional Scaling (NMDS). **(C)** UniFrac distance analysis. Boxes are the interquartile range; median values are bands within the boxes; whiskers are 1.5 times the IQR; and open circle is an outlier value. There would be statistic difference between two groups when the intergroup distance is evidently higher than the within-group distance. *p < 0.05 and **p < 0.01 versus the distance of “Model versus Model” (*n* = 8).

β-Analysis was used to compare the similarity of overall community structure, which employed several unsupervised multivariate statistical assessments, including PCA and UniFrac NMDS. Both PCA (Figure 8A) and UniFrac NMDS (Figure 8B) displayed a marked structure shift in samples of Model group in contrast to those of Normal group; while after the 3-week treatment, gut microbiome from the tumor-bearing mice of ESGH group was restored to be similar with that of Normal group. UniFrac distances analysis (both weighted and unweighted, Figure 8C) made a further confirmation on the structure remodeling by ESG, as indicated by the significant intergroup difference between Normal group and Model group, as well as that between ESGH group and Model group (*p* < 0.01).

#### Shifts in Community Membership After ESG Treatment

**Figure 9:**
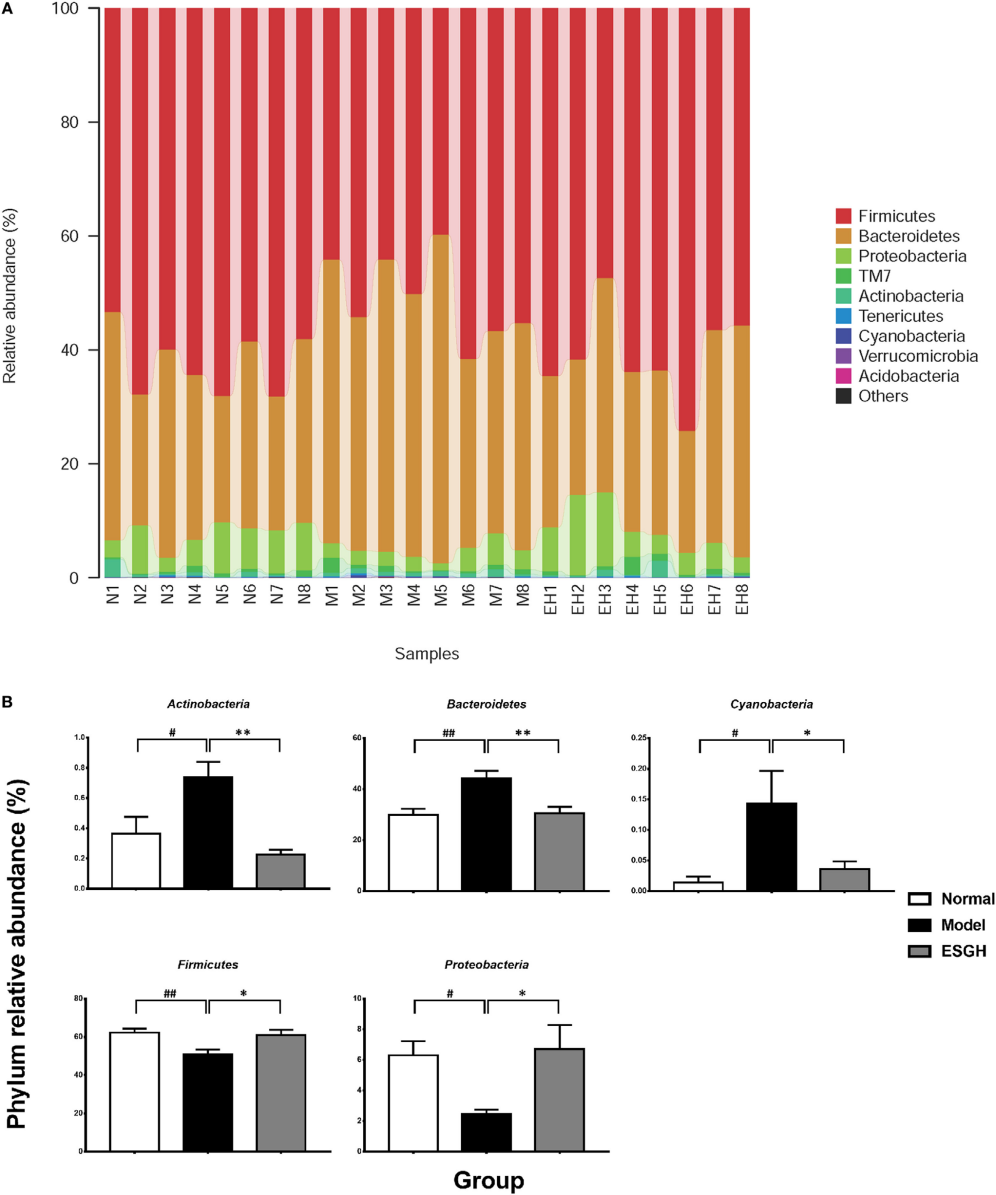
Taxonomy analysis on level of phyla. **(A)** Relative abundance of all detected phylum from each sample. **(B)** Significant intergroup differences were found in five phyla. Values were represented the means ± SEM (*n* = 8). ^#^*p* < 0.05 and ^##^*p* < 0.01 versus Normal group; *p < 0.05 and **p < 0.01 versus Model group.

**Figure 10:**
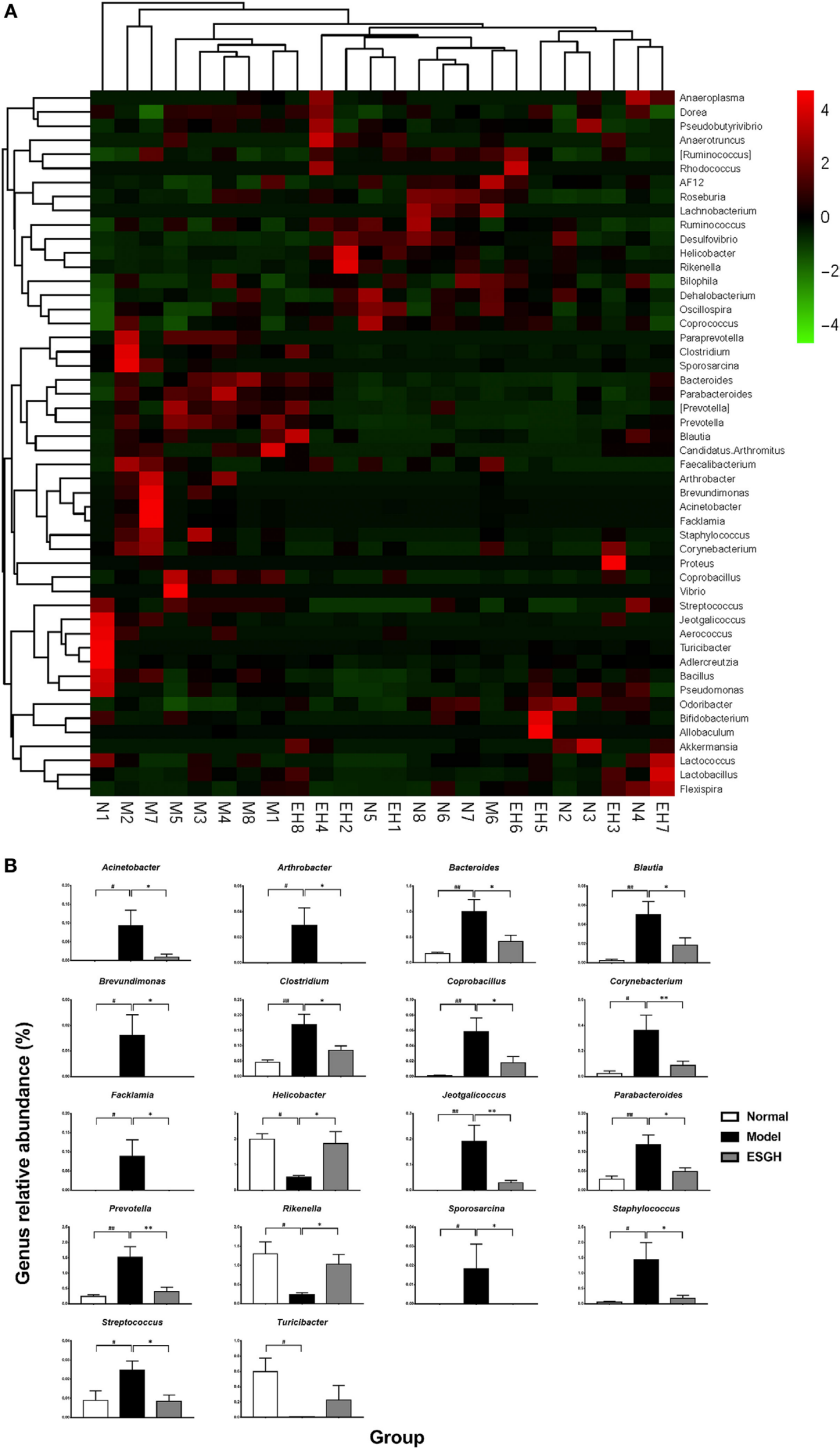
Taxonomy analysis on level of genera. **(A)** Heat map showing the relative abundance of major genera ranking top 50 from each sample. M, N, and EH denote Model group, Normal group, and ESGH groups, respectively. **(B)** Significant intergroup differences were found in 18 genera. Values were represented the means ± SEM (*n* = 8). ^#^*p* < 0.05 and ^##^*p* < 0.01 versus Normal group; *p < 0.05 and **p < 0.01 versus Model group.

Taxon-based analysis revealed marked differences at both phylum and genus levels among Normal, Model, and ESGH samples. Overall, a total of nine phyla were shared by samples from all groups (Figure 9A). Of them, *Firmicutes* and *Bacteroidetes* compromised over 90% of the total classified sequences. Relative abundances of *Actinobacteria, Bacteroidetes, Cyanobacteria, Firmicutes*, and *Proteobacteria* displayed significant differences in the three groups. In particular, ESGH treatment significantly raised the relative abundances of *Firmicutes* (*p* < 0.05) and *Proteobacteria* (*p* < 0.05) but reduced those of *Actinobacteria* (*p* < 0.01), *Bacteroidetes* (*p* < 0.01), and *Cyanobacteria* (*p* < 0.01), which apparently reversed the community shift induced by tumor xenograft (Figure 9B). At genus level, a total of 61 genera were identified from all samples (Data Sheet 2 in Supplementary Material), and hierarchical clustering analysis presented by heatmap showed that fecal microbiota from the Model group exhibited obvious community shift compared with those of Normal group and ESGH group (Figure 10A). Although LefSe analysis indicated no genus is specific for any group, there were 18 genus exhibited obvious differences among them. Briefly, three genera (*Helicobacter, Rikenella*, and *Turicibacter*) were evidently higher in both Normal group and ESGH group than those in Model group, which have been reported to be positively related to the enhanced immune response (47–50); while another 15 genera (*Acinetobacter, Arthrobacter, Bacillus, Bacteroides, Blautia, Brevundimonas, Clostridium, Coprobacillus, Corynebacterium, Facklamia, Jeotgalicoccus, Parabacteroides, Prevotella, Sporosarcina, Staphylococcus*, and *Streptococcus*) were lowered in both Normal group and ESGH group in contrast to those in Model group (Figure 10B).

#### Microbiome Function Regulation by ESG Treatment

**Figure 11:**
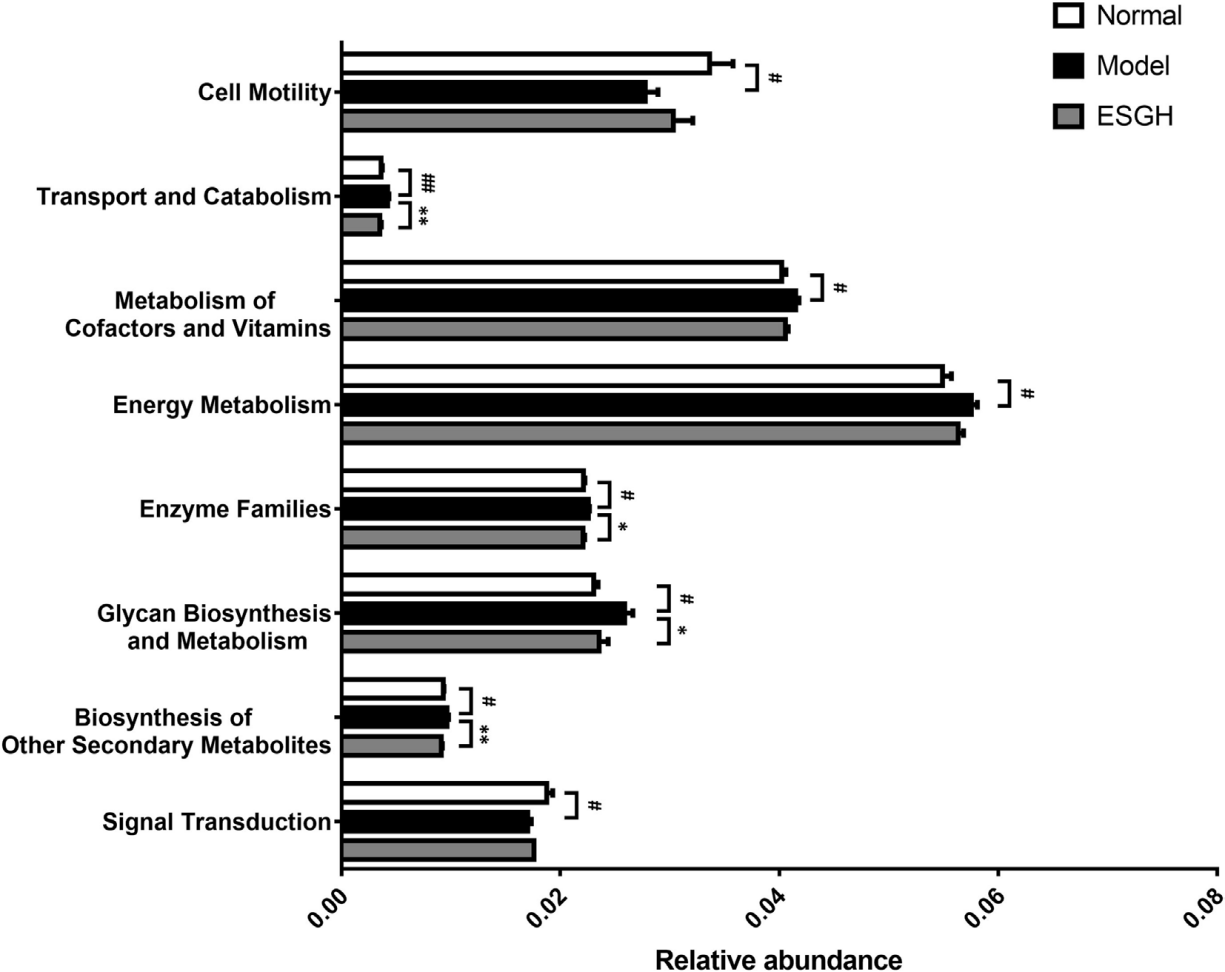
Microbiome function prediction according to Kyoto Encyclopedia of Genes and Genomes pathway database. Values were represented the means ± SEM (*n* = 8). ^#^*p* < 0.05 and ^##^*p* < 0.01 versus Normal group; *p < 0.05 and **p < 0.01 versus Model group.

*Via* comparing the sequencing data with those collected in KEGG pathway database by PICRUSt (Figure 11), it was found that tumor xenograft significantly upregulated abundances of several genes that are responsible for five metabolism pathways, including “biosynthesis of other Secondary metabolites,” “energy metabolism,” “enzyme families,” “glycan biosynthesis and metabolism,” and “metabolism of cofactors and vitamins,” and a cellular processes pathway (“transport and catabolism”), but downregulated genes involved in the “cell mobility” and “signal transduction” pathways. After ESGH treatment, changes in the genes that referred to most of these pathways were evidently reversed, such as “transport and catabolism,” “enzyme families,” “glycan biosynthesis and metabolism,” and “biosynthesis of other Secondary metabolites,” indicating that together with the structural modulation, ESG could regulate the metabolic activities of the gut microbiota to promote the tumor immune surveillance.

## Discussion

With the great success of paclitaxel in cancer chemotherapy, increasing attention has been paid for the natural compounds in prevention and treatment of cancer. So far, anticancer candidates derived from natural products, including alkaloids, saponins, polysaccharides, terpenoids, and flavonoids, have been extensively studied in laboratories and clinical investigations. More recently, potential of the active constituents from sporoderm-broken SG was also explored. Na et al. (30) found that the aforementioned polysaccharide exerted direct cytotoxicity on HCT116 *via* cell cycle arrest and apoptosis, but at a relative high concentration (1.25–7.5 mg/mL) (30); while Wang et al. (31) showed that a commercial polysaccharide from SG suppressed Sarcoma 180 only *in vivo via* the stimulation of NK cells, T cells, and macrophages (31).

In this study, anticancer potential of the polysaccharide-rich ESG was investigated in a murine 4T1-breast cancer xenograft model. Results showed that ESG effectively inhibited tumor growth both in terms of volumes and mass, accompanying with a significant necrosis in the tumor tissue; whereas this suppression was not mediated by a directly cytotoxicity as proved by the *in vitro* experiment. Numerous studies have demonstrated the importance of adaptive T cell-mediated cytotoxic responses as a dominant mechanism of host antitumor immune responses (51, 52), and TILs nesting in and around neoplastic cells have showed potential clinical implications, especially that the presence of CD8^+^ TIL is strongly associated with favorable prognosis in every solid human cancer studied virtually (53). Specifically, breast cancer patients with higher numbers of CD8^+^ TIL in the tumors are more likely to gain better outcomes concerning survival (54, 55). Tc cells (CD8^+^ T cells) are the most effective elements for tumor destruction (56). Spontaneously, these antitumor effector cells are first activated by the tumor cell-expressing surface molecules, such as calreticulin, tumor antigens in context of MHC class I molecules, and/or NKG2D ligands; then they lead apoptosis or inhibition on proliferation and angiogenesis to destroy the tumor tissue, mainly *via* increasing the cytotoxic factors within TME (including perforin, granzymes, IFN-α/β/γ, IL-1, IL-12, and TNF-α) (57). Despite not being an antitumor enforcer, Th cells (CD4^+^ T cells) have multiple impacts on the antitumor effect of Tc cells depending on the specific functions of various subsets, such as Th1, Th2, and regulatory Th cells (Treg). By secreting interferon-γ, Th1 cells are essential for the activation of Tc cells and have been shown to correlate with favorable survival in breast cancer (58). However, Treg cells are able to dampen the immune system so as to limit excessive immune responses that can cause collateral damage to normal tissue, which, on the other hand, resulted in a weaken on the antitumor function of Tc cells (58–61). In view of this, we hypothesized that the tumor control of ESG would be a restoration on the T cell paradigm. FCM data indicated that in the peripheral blood, percentage of Tc cells was increased by ESG treatment, and consequently, the ratio of Tc/Th was increased on account of the unchanged total T cell proportion. Intriguingly, CD8^+^ TILs of the tumor-bearing mice, together with the ratio of Tc/Th with TME, displayed a correspondent increase in response to ESG treatment. Moreover, it was found that ESG evidently increased the production of TNF in serum of the tumor-bearing mice but did not affect any other cytokines that are characteristic for Th1, Th2, or Th17 (Figure S4 in Supplementary Material). These collective results verified our hypothesis that by promoting the differentiation of T cell toward Tc, ESG employed Tc cells to potentiate the tumor immune surveillance, thereby effectively suppressing tumor growth. However, although ESG did not affect neither Th cell proportion in peripheral blood nor that in TME, its impact on Th cell subsets would be explored in the future due to their possible influence in tumor immune surveillance.

As depicted by the “3E immunoediting” theory, despite host antitumor immune responses stand the very heart of self-surveillance, the spontaneous tumor immunity, especially the dominant T-cell response, would not only be seized up by the tumor cell-driving “escape strategy” but also be limited in the setting of standard treatments (10, 52). Since derived from normal cells, tumor cells can develop several survival features to get immune tolerance, including reduced tumor antigens (CA15-3, CEA, PCNA, etc.), increased resistance or survival (STAT-3 or anti-apoptotic molecule Bcl2), or development of an immunosuppressive TME (releasing cytokines such as VEGF, TGF-β, or expressing immunoregulatory molecules such as IDO, PD-L1, Tim-3/galectin-9, and CTLA-4/CD80) (57). On the other hand, T-cell paradigm turns out to be anergy and exhaustion during cancer progression, due to the activation of membrane co-inhibitory signaling pathways, such as PD-1/PD-L1, and CTLA-4/B7 (11, 12), which finally results in decreased effector function and proliferative capacity. As demonstrated by our data, PTX apparently inhibited PD-1 and CTLA-4 in spleen but upregulated PD-1 in the tumor, implying a possible induction of anergy on the tumor immune surveillance. By contrast, ESG could evidently reduce the mRNA levels of *pd1* and *ctla4*, both in tumor tissue and spleen. Although effect of ESG on the protein expressions was not exactly consistent with that on mRNA level, it was also found that protein expression of PD-1 was significantly suppressed in tumor, whereas that of CTLA-4 was only downregulated in spleen.

Indeed, growing evidence reveals the correlation between mRNA and protein abundances in the cell is notoriously poor (62). This is because there exists the translational control on the process of protein translation from mRNA, involves mRNA degradation induced by microRNA and siRNA, initiation codon scanning, ribosome assemble, and so on, and the initiation phase is often the most regulated part (63–65). Thus, translational control results in an unequal protein translation from the transcribed mature mRNA, which enables the organisms to adapt to the changed circumstances *via* a quick regulation on the protein biosynthesis in each cell. As showed in the data, when compared with the normal control, protein level of PD-1 in spleen was reduced in the tumor-bearing mice, although the mRNA expression of PD-1 was upregulated. By contrast, PD-1 protein of ESG groups was evidently decreased, while their mRNA levels were equal to that of normal control group. Similar poor correlation between the mRNA and protein levels of PD-1 also exhibited in tumor. These results suggested that ESG would make profound regulations on the translation control of PD-1 and CTLA-4, so as to suppress this immune checkpoints signaling. Together with the previous results, it would be rationally to speculate that ESG could effectively restore the T cell paradigm by reversing the anergy and exhaustion status *via* suppressing the co-inhibitory checkpoints, thereby resulting in a favorite control on breast cancer. Furthermore, impacts on the tumor immune surveillance, such as co-inhibitory signaling pathway interaction (PD-1/PD-L1 and CTLA-4/CD86) and tumoral immunological balances (such as CD28: B7 binding versus CTLA-4: B7 binding), would be explored to make a more comprehensive exploration for the antitumor activity of ESG.

One more issue to be noteworthy is that, ESG did not exhibit obvious promotion on Tc cells differentiation *in vitro*. As ESG was administrated orally in this work, it is highly probable that a regulation on gut microbiota by ESG promotes the restoration on the exhausted Tc cells. Besides the cardinal role in the development and efficiency of immune system, microbiota also interact with gut mucosal surfaces thereby intervene the therapeutic responses for tumors occurring outside of the intestinal tract (16, 66). Specifically in breast cancer, increasing data have shown that gut microbiome is involved with all potential related factors of breast cancer, including immune regulation, metabolism of endogenous and exogenous substances, obese status, and so forth. CD8^+^ TIL has been found to be positive related to a better outcomes concerning survival in breast cancer patients (54), and an optimization on the gut microbiota community brings about a promotion on CD8^+^ T cell-mediated immunity, thereby indicating a better prognosis and an effective outcome of immunotherapy (14, 67). As demonstrated by our data, in despite of the reduced richness in the microbiome of the tumor-bearing mice (α-diversity), mice receiving ESG treatment shared a parallel microbiome structure as those of the normal counterparts when compared with the mice of Model group (β-diversity). Furthermore, ESG was able to reverse the microbiota community shift caused by tumor xenograft, of which 5 phyla (*Actinobacteria, Bacteroidetes, Cyanobacteria, Firmicutes*, and *Proteobacteria*) and 18 genera were significantly affected. Among the influenced genera, *Helicobacter, Rikenella*, and *Turicibacter* were the enriched ones that accounted over 0.5%, and especially, both *Helicobacter* and *Rikenella* were restored up to about 1%. Despite as pathogenicities to certain inflammatory-related carcinoma and autoimmune disorders, it has been revealed that *Helicobacter* and *Rikenella* were positively correlated to enhanced immune response (47, 48). *Turicibacter* was also strongly associated with immune functions, as indicated by the fact that *Turicibacter* populations within the gastrointestine would be almost abolished both in innate and adaptive immunodeficiency mouse models (49, 50). On the other hand, *Bacteroides* and other 14 genera were declined by ESG treatment. Typically, *Bacteroides* took up the biggest population (1% or so) among them, and it has been considered as a beneficial commensal when retained to a proper quantity in the gut, otherwise inducing bacteremia and abscess formation in multiple body sites (68). Nevertheless, increased abundance of *Bacteroides* is associated with immunosuppression (69) and carcinogenesis (70). Overall, ESG made a beneficial alterations on the gut microbiome community, i.e., to enrich the immunocompetence-related genera (*Helicobacter, Rikenella*, and *Turicibacter*) and to decline certain immunosuppressive-related ones (such as *Bacteroides*), resulting in a restoration on the dysbiosis induced by cancer xenograft.

Indeed, gut microbiota not only experiences long-term coevolution with host (71) but also influences the host immunity (72, 73). Especially, lifestyle, including diets and medicine intake, contributes greatly to the modification on gut microbiota (71). On the other hand, microbial utilization of complex polysaccharides is a major driving force in shaping the composition of the human gut microbiota (74, 75). In our works, it was found that ESG is mainly composed of polysaccharide made up of glucose, and it restored several pathways involving metabolism (“biosynthesis of other Secondary metabolites,” “energy metabolism,” “enzyme families,” “glycan biosynthesis and metabolism,” and “metabolism of cofactors and vitamins”), cellular processes (“transport and catabolism” and “cell mobility”), and environmental information processing (“cell mobility”). So it is probable that by oral administration, ESG was able to directly affect the growth homeostasis within the microbiota *via* regulating its metabolism mode, and possibly, serves as selective growth promoters to certain species. Therefore, microbiota community of the ESG-treatment samples was evidently shifted with lowered community richness (with reduced Chao1 and ACE indexes) but unaltered diversity. Nevertheless, the specific organisms affected by ESG have to be figured out and confirmed *via* experiment on germ-free animal models. Moreover, several studies revealed that natural polysaccharides would take several weeks to influence the gut microbiota (76, 77). It is probable that the remodeling by ESG may take several days to shift the community membership and to change the metabolism characteristics. Hence, tumor growth was not suppressed until the tumor immune surveillance was powerful enough by a long-term ESG treatment. Taken together, given that the promotion by ESG on Tc-mediated tumor suppression would only achieve by oral administration, it is feasible that the modulation on gut microbiome community would contribute greatly to ESG’s tumor control by restoring the exhausted Tc cells.

Of course, there are some more affairs to be taken in account for the antitumor activity of ESG, such as oxidative stress and aerobic metabolism (also namely Warburg effect). Oxidative stress has a controversial association with cancer, which is mainly mediated by reactive oxidative species (ROS). The elevated levels of ROS not only increases gene mutations and genomic instability (78, 79) but also inactivates the phosphatases within cancer cells, so their target proteins responsible for proliferation are kept activated, resulting in an uncontrolled cell growth (80). In these view of oxidative stress, Chikara et al. have reviewed the antioxidant potential of phytochemicals in cancer chemoprevention and treatment (81). However, activation of oxidation (such as by increasing ROS) contributes to autophagy and apoptosis in cancer cells, even though it is crucial for normal-to-cancerous cell transformation and cancer development (82, 83). Therefore, whether the antioxidant effect contributes to the anticancer activity depends on the various types of compounds. For instance, it is reported that ganoderic acids induce apoptosis in human cervical cancer HeLa cells *via* increasing the generation of intracellular ROS (84); while a *G. lucidum* extract elicited antitumor effects by suppressing cell growth and inducing antioxidative/detoxification activity in human ovarian OVCAR-3 cells (85). Regarding Warburg effect, it is indeed a metabolic nature of TME shifting from oxidative phosphorylation to aerobic glycolysis (86). It is critical for cancer progress and immune escape and characterized by increased glucose uptake and accumulation of lactate, accompanying with the upregulations of transporters, glycolytic enzymes, and the responsible signaling pathway proteins (87, 88). Therefore, pathways and activities involved in Warburg effect have been considered as novel targets in cancer therapy, such as metformin that acts via reducing the expression of monocarboxylate transporter 4 on cancer-associated fibroblasts (89). Although no researches report the impact on Warburg effect, it has revealed an antioxidant effect of the spore of *G. lucidum* in model of ischemia/reperfusion and streptozotocin-induced neuron damage (90, 91). In the future, we would pay attention to the possible role of antioxidant effect (or Warburg effect) by ESG in its anticancer activity.

Collectively, this study revealed that a polysaccharide-rich extract from sporoderm-breaking spore of *G. lucidum* (ESG) would serve as natural candidate for breast cancer treatment. The underlying mechanism of ESG was probably contributed to a suppression on co-inhibitory signaling (PD-1 and/or CTLA-4) and a consequent restoration on the exhausted Tc cells, to which the gut microbiome remodeling made a great contribution. Especially, such regulation involved not only changes in microbiota structure and community membership but also alternations in several metabolism pathways within the microbiome. So far, it has been found that, there is an intricate relationship between the biology of T cells (mainly regulatory T cells and Tc cell), and the various metabolites produced by host and commensal microbes, such as vitamins and short chain fatty acids (67, 92). Hence in all probability, regulation on the metabolism of gut microbiota would be the pivotal of ESG to enhance the Tc-mediated tumor surveillance against breast cancer. On the other hand, toxicity test is demanded to make comprehensive evaluation on its safety, although no article has yet reported the adverse effect of the spore of *G. lucidum*, except one suggests that an extract from fruit body of *G. lucidum* has lethal and sub-lethal effects on zebrafish embryos (93). And rigor pharmacokinetics study, as well as multi-omics investigations, for ESG will be applied to intercept the underlying mechanism of its immune-regulation activity, as well as the potential in cancer treatment.

## Ethics Statement

The experiment was performed according to the Guidelines of Guangdong Institute of Microbiology Laboratory Animal Center, Guangdong Institute of Microbiology Laboratory Animal Ethics Committee. The experimental protocols were approved by the Guangdong Institute of Microbiology Laboratory Animal Ethics Committee.

## Author Contributors

Conceived and designed the experiments; drafted and revised the manuscript: JS, YL, and YX. Performed the experiments: JS, LS, DL, OS, YZ, HL, and ZX. Analyzed the data: JS, LS, DL, and CJ.

## Conflict of Interest Statement

All coauthors declare that the research was conducted in the absence of any commercial or financial relationships that could be construed as a potential conflict of interest.

## Footnotes

^1^http://qiime.org/index.html (Accessed: July 25, 2017).

^2^http://qiime.org/scripts/pick_otus.html (Accessed: July 25, 2017).

^3^http://www.r-project.org (Accessed: July 25, 2017).

^4^http://kiwi.cs.dal.ca/Software/STAMP (Accessed: July 25, 2017).
