# Supplementary material for: Antitumor Activity of Extract From the Sporoderm-Breaking Spore of Ganoderma lucidum: Restoration on Exhausted Cytotoxic T Cell With Gut Microbiota Remodeling
Source: Front Immunol. 2018 Jul 31;9:1765. doi: 10.3389/fimmu.2018.01765 (PMC6079217; doi:10.3389/fimmu.2018.01765)
Supplement: Supplementary file 1 [file Data_Sheet_1.DOCX]

**Materials and methods.**

1. *Preliminary characteristics of ESG*
   1. *Determination of* *relative molecular mass*

Relative molecular mass of polysaccharide was determined according to the method of size-exclusive HPLC. HPLC (LC-20A, SHIMADZU), equipped with a refractive index detector (RID) and a series of guard colum, TSK-Gel G5000PWXL column, and TSK-Gel G4000PWXL column (Tosoh Corp. , Tokyo), was used in this test. Sample was disolved in 50mM Na_2_SO_4_, and eluted with 50mM Na_2_SO_4_ at a flow rate of 0.5 m L/min. Temperature of column and detector was set at 35 ℃. Standard glucans (0.18 kDa, 5 kDa, 12 kDa, 25 kDa, 50 kDa, 80 kDa, 150 kDa, 270 kDa, 410 kDa, and 670 kDa) were used as references. Weight average molecular weight (Mw) was calculated with Agilent GPC/SEC software.

- 1. *Determination of monosaccharide composition*

Monosaccharide composition of polysaccharide was detected by the reported method. Sample (~5 mg) was dissolved in trifluoroacetic acid solution (2 M, 4 mL), and hydrolyzed at 120 ℃ for 2 h. The hydrolyzed product was evaporated to dryness and derivatized by the following method. Hydroxylammonium chloride (10 mg), inositol (5mg, used as the internal reference) and pyridine (0.6 mL) were added to the hydrolyzed sample. Mixture was placed in water bath of 90 ℃ and vibrated for 30min. After cooling, acetic anhydride (1 m L) was added, and then the reaction system was set in water bath of 90 ℃ and oscillated for 30 min again. The reaction products were analyzed in GC (7890B system, Agilent) with a flame ionization detector (FID) and a HP-5 capillary column (30 m × 0. 32 mm, 0.25 μm). The operation conditions of GC were as follows : flow rate N2, H2 and air was 1 m L / min, 30 m L / min and 400m L / min, respectively; make up flow was 25 mL/min; the temperature of detector and inlet was 250 ℃ and 250 ℃, respectively; the oven temperature program was set changing from 120 ℃ (standing for 3 min) up to 180 ℃ (standing for 8 min ) at a rate of 2 ℃/min, and then up to 210 ℃ (standing for 4 min ) at a rate of 2 ℃/min. Standard monosaccharides (rhamnose, arabinose, fucose, xylose, mannose, glucose and galactose) were also derivatized and analyzed in GC as references.

1. *Effect of ESG on splenocytes in vitro*
   1. *Cell preparation.*

Balb/c mice were sacrificed, and the spleens were harvested and ground in pre-cold PBS by passing through a 70μm strainer. After centrifuge at 300×*g* for 5 min at 4℃, erythrocytes were lysed in ACK Lysis Buffer for 5 min at room temperature. Cells were collected by centrifuge at 300×*g* for 5 min at 4℃, and washed twice with pre-cold PBS. Then cells were resuspended in complete RPMI1640 containing 10% heat-inactivated FBS, and 100 IU/mL of penicillin and streptomycin for the following experiment. Cell viability was determined by trypan-blue dye exclusion, and cell viability was higher than 95 % in all cases. Cell culture was maintained in a humidified atmosphere of 5 % CO_2_ at 37 °C.

- 1. *Population analysis of B cell, T cell, and T cell subsets*

Splenocytes were seeded at a density of 3 × 10^6^ cells/mL in 96-well plates (triplicate wells in each group), and treated with 100μL ESG in complete RPMI 1640 medium of multiple concentrations (0, 100, 200, 400 μg/mL) for 72 h. Cells were harvested and stained with FITC anti-mouse CD3, APC anti-mouse CD19, PE-Cy7 anti-CD4, PE anti-CD8 (eBioscience, Thermofisher Scientific, San Diego, CA) at 4 °C in dark for 30min. After washed twice with PBS, cells were resuspended in 2% paraformaldehyde and detected by FACS Canto II cytometer. The data was analyzed by Diva software (version 6.1.3).

1. *Serum cytokines determination*

Releases of cytokines in the serum, including IL-17A, TNF, IFN-γ, IL-6, and IL-4 were measured by Cytometric Bead Array (CBA) Mouse Th1/Th2/Th17 Cytokine Kit (BD, NY, USA) according to the manufacturer’s instruction. Briefly, peripheral blood was collected from the orbital vein plexus before tumor harvest. Serum was separated from the obtained blood by centrifuge (1000×*g*, 20 min). 25 μl of serum was incubated with the mixture of capture bead suspension and the PE detection reagent for 2 h. Samples were washed with PBS and detected by FACS Calibur flow cytometer, and data was analyzed by FCAP software (version 3.0, BD, NY, USA). Cytokine standards provided with the kit were appropriately diluted and used in parallel to samples for preparation of the standard curves.

**Results**

1. *Characteristics of ESG.*

Results showed that ESG had a Mw of 3659 Da (Table S 1 and Figure S 1), and it is mainly composed of glucose (Figure S 2)

Table S 1 Retention time table of standard glucans and ESG

| Sample name | Retention time/min |
| --- | --- |
| 180 Da | 45.228 |
| 5000 Da | 42.171 |
| 12000 Da | 40.364 |
| 25000 Da | 38.180 |
| 50000 Da | 36.228 |
| 80000 Da | 34.770 |
| 150000 Da | 33.147 |
| 270000 Da | 31.735 |
| 410000 Da | 30.671 |
| 670000 Da | 30.094 |
| ESG | 44.417 |

Figure S 1 Size-exclusive HPLC spectrum of ESG.


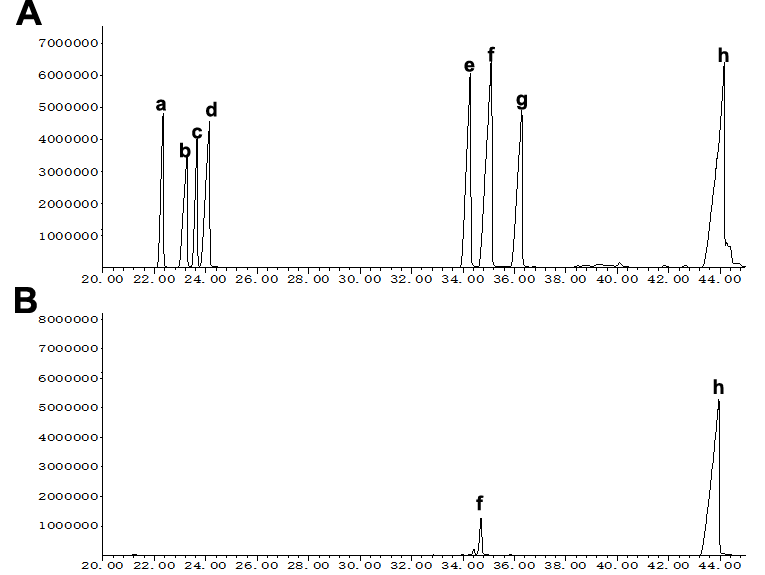


Figure S 2 GC spectra of monosaccharide reference (A) and monosaccharide composition of ESG (B). a. Rhamnose; b. Arabinose; c. Fucose; d. Xylose; e. Mannose; f. Glucose; g. Galactose; h. Inositol.

1. *ESG did not directly stimulate splenocytes in vitro.*

As indicated in Figure S 3, populations of B cell (CD^19+^), T cell (CD^3+^), helper T cell (CD^3+^CD^4+^), and cytotoxic T cell (CD^3+^CD^4+^) in the total splenocytes did not affected after the stimulation of ESG for 72 h.


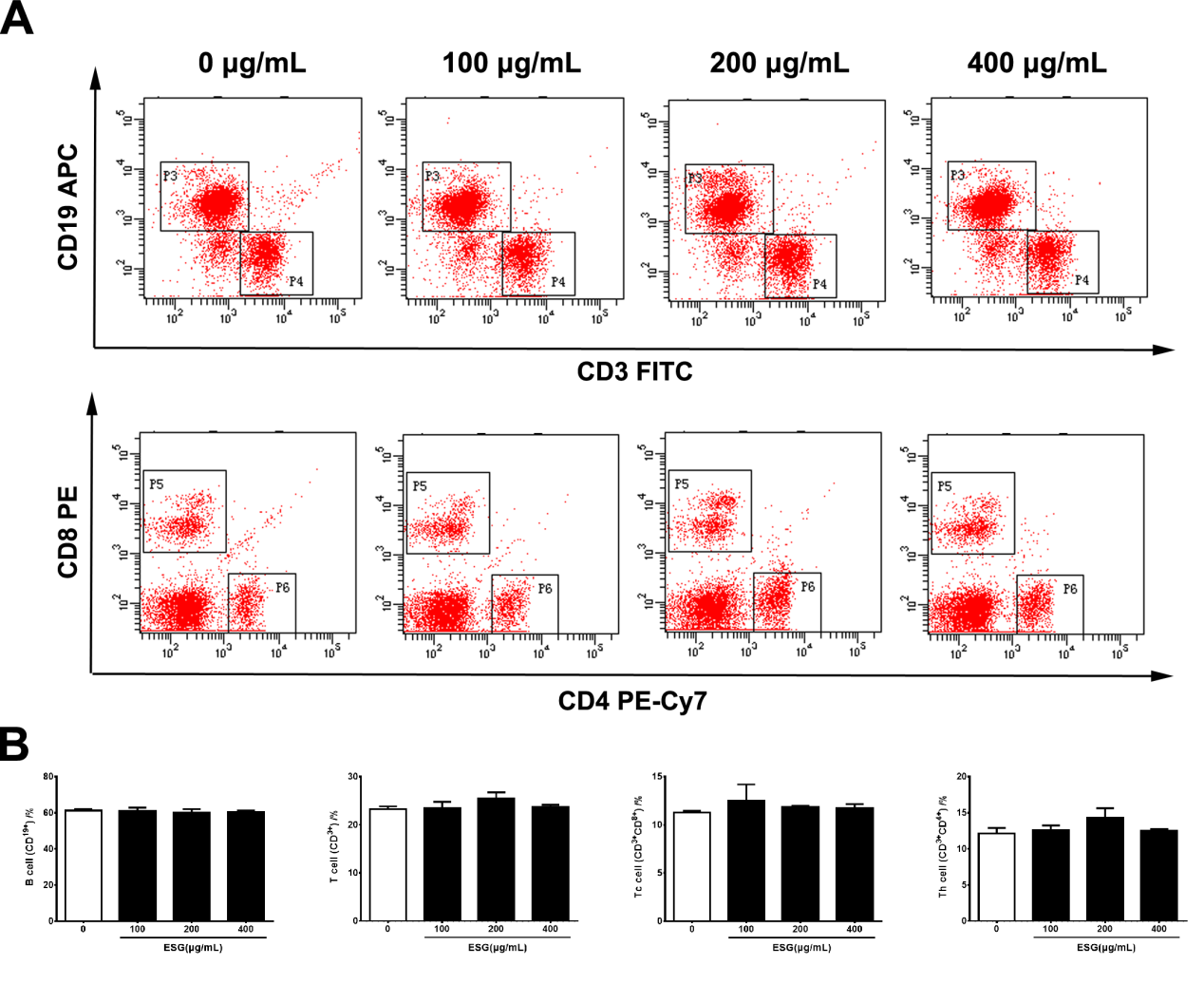


Figure S 3 Effect of ESG on splenocytes *in vitro*. (A) Representative scatter diagram. (B) Quantitative analysis for populations of B cell, T cell, and the T cell subsets in splenoctyes. Values were represented the means ± SEM (*n*= 3). # *p* < 0.05 and ## *p* < 0.01 versus Normal group.

1. *ESG posed various impacts on the serum cytokines in tumor-bearing mice*

Results were showed in Figure S 4. IL-4 is a symbol of Th2 cells, but it could be hardly determined in most of the samples, except that it was extremely high in the samples of PTX group (*p* < 0.01). IFN-γ and IL-17A are symbols for Th1 and Th17 cell, respectively. Although tumor xenograft did not make evident changes on these two cytokines, PTX treatment evidently increased the release of IFN-γ (*p* < 0.01), but ESG treatment did not affect neither of them. IL-6 and TNF are typical inflammatory cytokines that would contribute to tumor destruction. As depicted by the data, IL-6 was significantly lifted while TNF was suppressed in Model group (*p* < 0.01). By contrast, both PTX and ESG promoted the production of TNF (*p* < 0.01), but did not affected that of IL-6.





Figure S 4 Effect of ESG on serum cytokine. Values were represented the means ± SEM (*n*= 6~8). # *p* < 0.05 and ## *p* < 0.01 versus Normal group.
